# Sources of Postacute Care Episode Payment Variation After Traumatic Hip Fracture Repair Among Medicare Beneficiaries: Cross-Sectional Retrospective Study

**DOI:** 10.1097/AS9.0000000000000218

**Published:** 2022-11-07

**Authors:** John R. Montgomery, Pooja U. Neiman, Craig S. Brown, Anne H. Cain-Nielsen, John W. Scott, Naveen F. Sangji, Bryant W. Oliphant, Mark R. Hemmila

**Affiliations:** From the * Center for Healthcare Outcomes & Policy, Department of Surgery, University of Michigan, Ann Arbor, MI; † National Clinician Scholars Program, University of Michigan, Ann Arbor, MI; ‡ Department of Surgery, Brigham and Women’s Hospital, Boston, MA; § Department of Orthopaedic Surgery, University of Michigan, Ann Arbor, MI.

**Keywords:** geriatric trauma, hip fracture repair, payment variation

## Abstract

The objective of this study was to evaluate how much variation in postacute care (PAC) spending after traumatic hip fracture exists between hospitals, and to what degree this variation is explained by patient factors, hospital factors, PAC setting, and PAC intensity.

**Background:**

Traumatic hip fracture is a common and costly event. This is particularly relevant given our aging population and that a substantial proportion of these patients are discharged to PAC settings.

**Methods:**

It is a cross-sectional retrospective study. In a retrospective review using Medicare claims data between 2014 and 2019, we identified PAC payments within 90 days of hospitalization discharges and grouped hospitals into quintiles of PAC spending. The degree of variation present in PAC spending across hospital quintiles was evaluated after accounting for patient case-mix factors and hospital characteristics using multivariable regression models, adjusting for PAC setting choice by fixing the proportion of PAC discharge disposition across hospital quintiles, and adjusting for PAC intensity by fixing the amount of PAC spending across hospital quintiles. The study pool included 125,745 Medicare beneficiaries who underwent operative management for traumatic hip fracture in 2078 hospitals. The primary outcome was PAC spending within 90 days of discharge following hospitalization for traumatic hip fracture.

**Results:**

Mean PAC spending varied widely between top versus bottom spending hospital quintiles ($31,831 vs $17,681). After price standardization, the difference between top versus bottom spending hospital quintiles was $8,964. Variation between hospitals decreased substantially after adjustment for PAC setting ($25,392 vs $21,274) or for PAC intensity ($25,082 vs $21,292) with little variation explained by patient or hospital factors.

**Conclusions:**

There was significant variation in PAC payments after a traumatic hip fracture between the highest- and lowest-spending hospital quintiles. Most of this variation was explained by choice of PAC discharge setting and intensity of PAC spending, not patient or hospital characteristics. These findings suggest potential systems-level inefficiencies that can be targeted for intervention to improve the appropriateness and value of healthcare spending.

## Abstract 2

Mini-Abstract: We analyze the primary drivers of variation in postacute care payments after traumatic hip fracture repair among Medicare beneficiaries. We find that mean unadjusted postacute care payments varied by $14,150 per episode between the highest- and lowest-spending hospital quintiles but decreased substantially after adjustment for postacute care setting and intensity.

## INTRODUCTION

Traumatic hip fracture is a common and costly acute medical condition, particularly among Medicare beneficiaries.^1^ The most prevalent mechanism of injury resulting in a trauma center admission is a fall (47%).^2,3^ Isolated hip fracture cases comprise 12% of trauma admissions in the United States and the incidence of hip fracture cases among patients age ≥65 years old is >300,000 patients annually and growing.^4–6^ Postacute care (PAC) spending has been identified as the fastest growing major spending category and is the component of care responsible for the widest variation in per capita Medicare spending.^1,7,8^

PAC payments following traumatic injury comprises the greatest proportion (48%) of total 90-day episode payments among fee-for-service Medicare beneficiaries, outweighing the proportions spent on index hospitalizations (40%) or readmissions (11%).^9^ With the anticipated growth in incident trauma cases and healthcare spending among the aging population, understanding the drivers of PAC payments among Medicare beneficiaries with traumatic hip fracture is crucial.^10^ Discharge destination decisions, including the type and intensity of care received, can be driven by clinical and nonclinical factors such as provider availability, geographic location, and Medicare methods of payment.^11^

In this context, we leveraged national Medicare data to identify sources of PAC spending variation after traumatic hip fracture. First, we investigated to what degree payment varies by patient-level characteristics. Second, we sought to determine what hospital-level factors were associated with increased variation. Finally, we explored what variation was explained by PAC setting and intensity. We hypothesized that the choice of PAC discharge setting and intensity of PAC spending would explain more variation in total PAC spending than patient case-mix and hospital factors.

## METHODS

### Data Source and Study Population

We used Centers for Medicare and Medicaid Services MedPAR (Medicare Provider Analysis and Review file for hospital and skilled nursing stays), Carrier, Outpatient, and Home Health Agency claims data. We selected for claims after hip fracture injury among fee-for-service Medicare beneficiaries ≥65.5 years old from October 1, 2014, to September 30, 2019, using a nationally representative 20% sample. The age cutoff of 65.5 years old was chosen to permit adjustment for healthcare expenditures within the preceding 6 months before traumatic injury. Patients with third-party insurers (eg, Medicare Advantage plans) were excluded from analysis. These data were linked with the American Hospital Association (AHA) Annual Survey of Hospitals from 2016 to obtain hospital-level information.

Beneficiaries with a primary diagnosis of hip fracture were identified using International Classification of Disease (ICD) 9 and ICD-10 diagnosis codes (see Supplemental Table, http://links.lww.com/AOSO/A180, which includes the ICD-9 and ICD-10 codes used to create the traumatic hip fracture injury cohort). ICD diagnosis codes listed in the Centers for Medicare & Medicaid Services Comprehensive Care for Joint Replacement model were matched with Abbreviated Injury Scale (AIS) codes defining the acute hip fracture injury analysis cohort published by the American College of Surgeons Trauma Quality Improvement Program.^12–14^ We excluded ICD-9 and ICD-10 codes that identified patients with pathologic, atypical, or acetabular fractures as well as subsequent encounters for delayed healing, nonunions, or malunions. AIS values for the severity of injury were mapped from claim primary and secondary ICD-9 and ICD-10 diagnosis codes via open-access R software (ICDPIC-R).^15^ An Injury Severity Score (ISS) was calculated from the AIS severity of injury values for each patient.

**FIGURE 1.**
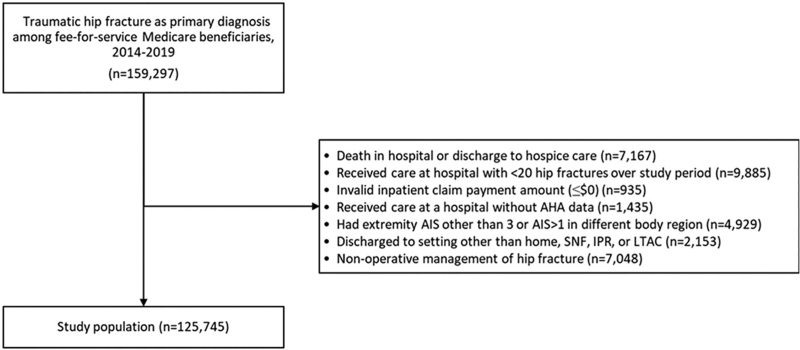
Flow diagram of study inclusion and exclusion criteria.

Observations were excluded if any of the following criteria were met: any AIS extremity value other than 3 (not consistent with isolated hip fracture); AIS value greater than 1 in any other body region (indicative of polytrauma); nonoperative management of traumatic hip fracture; death during index hospitalization or discharge to hospice; care at a hospital with less than 20 hip fracture diagnoses over the 5-year study period; invalid inpatient claim amount (≤$0); or care at a hospital without AHA data. A flow diagram of study inclusion and exclusion criteria can be found in Figure 1.

### Primary Outcome

Our primary outcome was total PAC payments by episode within 90 days of index hospitalization discharge. We defined PAC payments as the presence of claims for any of these types of services within 90 days of index hospitalization discharge: home with home health, skilled nursing facility (SNF), inpatient rehabilitation (IPR), outpatient rehabilitation, and long-term acute care (LTAC) facility. If patients were readmitted, the payments associated with readmission were included in the primary outcome measures. Each of these settings have different payment models that are important to acknowledge.^16^ Skilled nursing facilities, outpatient rehabilitation, and LTAC facilities are reimbursed based on a per-diem basis with various adjustments according to the patient’s clinical status, functional status, and services provided. Conversely, home health services and inpatient rehabilitation are reimbursed on a per-case basis with similar adjustments so long as minimum services are provided. All payments were inflation-adjusted to 2019 US dollars ($USD).

### Statistical Analyses

Our goal was to determine the drivers of differences in mean episode PAC payments by hospital quintile. First, we investigated whether patient demographics, injury patterns, and hospital characteristics were systematically different between PAC spending quintiles. Hospitals were placed into quintiles based on their inflation-adjusted (2019 $USD) total mean PAC spending per episode. These characteristics were compared with χ^2^ test for categorical variables or analysis of variance F-test for continuous variables.

Next, we compared the distributions of discharge destination (home, home health, skilled nursing facility, inpatient rehabilitation, and LTAC facility) across hospital quintiles of total PAC spending. We then measured how each component of PAC spending (skilled nursing facility, inpatient rehabilitation, home health, readmissions, outpatient rehabilitation, LTAC, plus total PAC spending) differed across hospital quintiles of PAC spending.

Finally, we assessed the degree to which mean episode PAC payment variation was explained by differences in geographic location, patient characteristics, hospital characteristics, readmissions, and choice and intensity of PAC setting across hospital quintiles. Sequential adjustment was performed to quantify PAC payment variation explained by the following factors: (1) price standardization; (2) patient characteristics (age, sex, race, Elixhauser comorbidities,^17^ injury severity score, complications during index hospitalization defined according to the Complications Screening Project that included pulmonary failure, pneumonia, myocardial infarction, venous thromboembolism, renal failure, hemorrhage, surgical site infection, and gastrointestinal bleeding,^18,19^ index hospitalization inpatient spending, and total prior 6-month spending); (3) hospital characteristics (bed size, teaching status, for-profit status, certified trauma center status, rural versus urban, critical access status, and annual traumatic hip fracture operative volume modeled a priori as a continuous variable with splines at 10 and 30 episodes per year in the study’s 20% representative sample); (4) readmissions; and (5) either choice of PAC setting or intensity of spending (the amount of spending within a PAC setting).

Price standardization was performed to account for intended differences in Medicare payments. These include compensation differences based on regional wage disparities, cost of living, illness severity, and the expense of caring for underinsured patients.^20^ We used price standardization methods previously employed within our research group.^8,20^ The methods have been described by researchers with the *Dartmouth Atlas of Healthcare* and are similar to the methods used by the Medicare Payment Advisory Commission.^21^ Sequential adjustments for patient characteristics, hospital characteristics, and readmissions were performed using multivariable regression models. Observed-to-expected ratios were calculated using expected values from these models. To measure the effect of choice of the PAC setting, the percentage use of a given PAC setting was held constant across observations, while allowing spending to vary. To measure the effect of intensity, spending within a given PAC setting was held fixed, while allowing percentage use to vary. After the sequential inclusion of each level, we calculated the difference in mean episode PAC payments between the highest (Q5) and lowest (Q1) hospitals, as well as the percentage difference in PAC payments between quintiles, measured as a share of the lowest-spending quintile (mean 90-day PAC payments between the highest- (Q5) and lowest (Q1)-spending quintiles, divided by the mean 90-day PAC payment of the lowest (Q1)-spending quintile: (Q5 – Q1)/Q1).

We assessed statistical significance at α = 0.05 with two-sided tests. All analyses were conducted using SAS 9.4 and Stata 15.0. The University of Michigan Institutional Review Board approved this study as secondary use of existing data with waiver of consent. The reporting guideline for cohort studies published by the Strengthening the Reporting of Observational Studies in Epidemiology (STROBE) was followed.^22^

## RESULTS

### Patient, Injury, and Hospital Characteristics and PAC Payments

**FIGURE 2.**
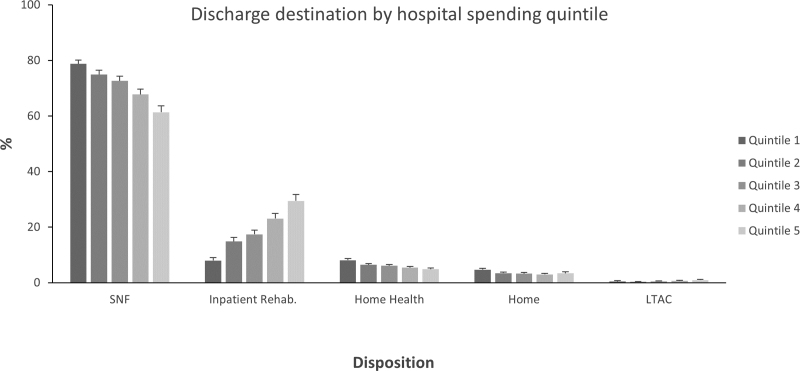
Discharge destination by hospital spending quintile. Hospitals were placed into quintiles based on their inflation-adjusted mean PAC spending per episode. These discharge destinations are shown as 5 components: home, HH, SNF, IPR, and LTAC. Error bars represent the 95% confidence interval. HH indicates home health

Of 159,297 traumatic hip fracture episodes identified, 125,745 (78.9%) met study inclusion criteria and received care at 2078 hospitals. Hospitals were placed into quintiles based on their inflation-adjusted mean PAC spending per episode. Patient, injury, and hospital characteristics are described for the lowest and highest payment quintiles in Supplemental Data File 2, http://links.lww.com/AOSO/A181. Significant differences were present between the highest- and lowest-spending quintiles hospitals for age, race, rate of inpatient complications, hospital length of stay, readmissions, and geographic region. The highest-spending hospitals were more likely to be teaching hospitals, have more hospital beds, and an urban location. Approximately 90% of fee-for-service Medicare beneficiaries who experienced a traumatic hip fracture were discharged to a PAC setting other than home, with variation across total PAC spending quintiles (Fig. 2). Although discharge to a PAC setting other than home was common among all hospital quintiles, mean PAC payments varied by $14,150 between the highest and lowest quintiles with a stepwise increase between all hospital quintiles (Table 1).

**TABLE 1.** Mean PAC Payments by Hospital Spending Quintile

Quintile	SNF	IPR	HH	OPR	LTAC	Readmit	Total
Q1	$11,336	$1276	$1894	$768	$63	$2343	$17,681
Q2	$12,668	$2573	$2001	$757	$160	$2963	$21,123
Q3	$13,721	$3279	$2036	$792	$222	$3371	$23,421
Q4	$14,357	$4662	$2196	$774	$311	$3800	$26,101
Q5	$16,804	$6685	$2357	$760	$529	$4696	$31,831

Hospitals are placed into quintiles based on their inflation-adjusted mean PAC spending per episode. These PAC payments are shown as 6 components—SNF, IPR, HH, OPR, LTAC, and readmissions (Readmit)—in addition to total PAC payments (Total).

HH indicates home health; OPR, outpatient rehabilitation.

### Impact of Price Standardization, Case-Mix, Hospital, and Readmission Adjustment on PAC Spending

Price standardization reduced the difference in mean PAC spending between Q5 (high spending) and Q1 (low spending) hospitals by $5186 (Table 2). The delta as a percent of mean quartile one PAC spending declined from 80% to 48.1% with price standardization. Patient, injury, and hospital characteristics explained 5% ($720) of the overall variation between quintiles. For example, the difference in PAC payments between the highest- and lowest-spending hospitals after price standardization was $8964 per episode. After risk-adjusting for patient, injury, and hospital characteristics, this difference decreased slightly to $8244 per episode. After adjusting for readmissions, this difference decreased to $6947 per episode. Index hospitalization inpatient spending was not correlated with PAC spending (ρ = −0.012, 95% confidence interval −0.057 to 0.033) after completion of risk-adjustment (Supplemental Figure, http://links.lww.com/AOSO/A182, which illustrates hospital-level mean risk-adjusted inpatient payments and mean risk-adjusted total 90-day PAC payments).

**TABLE 2.** Mean Episode PAC Payments in Dollars After Sequential Adjustment for Traumatic Hip Fracture Among Medicare Beneficiaries

	Total Payments, by Hospital Quintile		Q5 − Q1	
	Q1 (Low)	Q5 (High)	Dollars ($)	% of Q1
Actual payment (2019 $USD)^*^	17,681	31,831	14,150	80.0
(+) Price standardization	18,637	27,601	8964	48.1
(+) Case-mix adjustment	18,886	27,448	8582	45.5
(+) Hospital adjustment	19,023	27,267	8244	43.3
(+) Readmission adjustment	19,639	26,586	6947	35.4
(+) Setting *or* intensity adjustment				
Setting adjustment^†^	21,274	25,392	4119	19.4
Intensity adjustment^‡^	21,292	25,082	3790	17.8

Percentage of Q1 is calculated as the difference in mean 90-day PAC payments between the highest (Q5) and lowest (Q1) spending quintiles divided by the mean 90-day PAC payment of the lowest (Q1) spending quintile: (Q5 − Q1)/Q1.

^*^Payments inflation-adjusted to 2019 $USD.

^†^Setting adjustment involves fixing the proportion of patients discharged to a given PAC setting and then allowing PAC payments to vary by hospital.

^‡^Intensity adjustment involves fixing the payments by episode for each PAC setting and then allowing the proportion of patients discharged to that setting to vary by hospital.

USD indicates United States dollars.

### Type and Intensity of PAC Utilization

When we examined the sources of variation in inflation-adjusted mean PAC payments across hospital quintiles, an increase in IPR utilization (8 vs 29%) and decrease in SNF utilization (79% vs 61%) was observed when transitioning from the lowest- to highest-spending hospital quintile (Fig. 2). Discharge to home with or without home health was also more common (13%) among the lowest-spending hospital quintile, although this difference was less substantial (quintile 5 = 8%). After adjusting for price standardization, case-mix, hospital characteristics and readmissions, subsequent adjustment for PAC setting, or intensity resulted in substantial reductions in differences in mean PAC spending between hospital quintiles (Table 2). Adjustment for PAC setting decreased the difference to $4119 per episode, whereas adjustment for PAC intensity decreased the difference to $3790 per episode. Following adjustment for setting or intensity 17.8%–19.4% of the original variability in mean PAC payments between hospital quintiles remained unexplained.

## DISCUSSION

In this nationally representative study of Medicare beneficiaries with a primary diagnosis of traumatic hip fracture who underwent operative repair, we found that discharge to a PAC setting other than home is common, occurring in over 90% of cases. However, mean 90-day PAC payments vary substantially between treating hospitals. The highest-spending hospitals tended to discharge patients to IPR, whereas the lowest-spending hospitals tended to discharge patients to SNF settings. Patient, injury, and hospital characteristics explained little of the variation in PAC spending (4.8%), whereas setting or intensity of PAC explained most of the total variation (16%–17.6%) after price standardization in risk-adjusted payments.

If variation is largely driven by patient factors such as injury severity, demographic characteristics, and baseline comorbidities, then higher payments for sicker patients might be appropriate. However, substantial payment variations after adjusting for patient-level factors would suggest systems-level inefficiencies that can be targeted for intervention to improve the appropriateness and value of healthcare spending.^23–25^ Concern regarding the relationship between the variation in healthcare expenditures and the quality of care has led to recent collaborative quality initiatives focused on achieving high-quality care with the efficient use of scarce healthcare resources.^25–27^

We found that even after controlling for patient, injury, and hospital characteristics, substantial variation exists across hospitals in PAC spending and utilization of PAC services. The decision regarding which if any PAC setting to discharge a patient to following traumatic hip fracture is complex and often involves input from a multidisciplinary team, in addition to the patient and their family. The team includes physicians, physical therapists, occupational therapists, case managers, social workers, and others. Preferences may exist among these team members who favor particular PAC settings even with similar case-mix characteristics between hospitals. This may be influenced by improved outcomes according to PAC setting among other hospitalized patient populations that members of the multidisciplinary team also manage. For instance, patients with cerebrovascular accident have been shown to have better functional outcomes when discharged to IPR facilities.^28,29^ To our knowledge, no qualitative studies have investigated decision patterns among multidisciplinary teams regarding choice of PAC setting among traumatic hip fracture patients. There is also evidence that nonclinical unmeasured hospital characteristics drives the majority of the variation in discharge location for trauma patients.^30^ It is also uncertain what influence patient preferences have on the choice of PAC setting.

The choice of PAC setting or intensity has not been shown to influence functional outcomes among hip or lower extremity fracture cohorts, although these observational studies are likely limited by selection bias.^28,31–33^ Previous studies have suggested that recovery to pre-fracture activities of daily living (ADL) occurs in only 31% of patients following hip fracture and that a substantial portion (64%) of those with ADL independence before injury do not return to ADL independence following their acute care episode.^34^ Whether the higher costs associated with settings such as IPR add value to the care of patients with hip fracture and justify their higher costs remains to be seen.^32,35^

Hospital ownership of PAC facilities may also play a role in choice of PAC setting. According to a recent study by Carroll et al, 80% of AHA hospitals owned at least 1 PAC facility.^36^ This might incentivize discharge to one of these facilities. Interestingly, the authors found that PAC facility ownership was associated with lower episode payments for SNF and home health services but higher episode payments for IPR settings. In our study, hospital factors such as bed size and teaching status were not associated with substantial PAC payment variation between hospital quintiles. However, hospital ownership of PAC facilities was not available for analysis in our study and its contribution to PAC payment variation between hospitals will be an important area of future research.

Finally, it is unclear to what degree variability in PAC facility availability contributes to variation in spending. Certain geographic locations may suffer from poor accessibility to timely placement of patients into rehabilitation facilities following traumatic hip fracture and this may affect utilization patterns beyond the control of the hospitals which discharge these patients. Data from the 2020 Medicare Payment Advisory Commission Report to Congress show that nationwide inpatient rehabilitation average occupancy rates are near 67%, suggesting that overall occupancy is not a particular barrier to rehabilitation utilization, but that this may still be a factor in specific geographic locations.^37^

Our study has several important limitations. The principal limitation to our analyses is related to the inability to assess functional status and its interaction with PAC utilization. Medicare claims do not contain granular clinical information regarding the degree to which patients are functional either prior to or following their acute hospitalization and recovery. This information has implications for patient eligibility for and appropriateness of PAC services and limits our assessment of how variation in functional status may account for variation in PAC spending. We are not aware of any prior studies suggesting that significant variation in the baseline mobility or functional independence of patients across hospitals exists for hip fracture patients in the United States, and therefore would not expect this to explain a significant proportion of the variance in PAC spending. The lack of mobility or functionality data in the postinjury period also limits our ability to assess the degree to which variation in the utilization of PAC rehabilitation services improves outcomes in these patients, and therefore, we are unable to make any assessment of value of these services in this analysis. Our analysis is also limited to fee-for-service Medicare beneficiaries who underwent operative management for their injuries and therefore may not be generalizable to other populations with hip fracture such as those less than 65 years of age, Medicare beneficiaries with additional third-party insurers, or patients who do not undergo operative management for their injuries. Finally, we excluded patients with significant polytrauma (AIS > 1 to any other body region) and our results may not be generalizable to this population.

## CONCLUSIONS

This study demonstrates a substantial variation in PAC payments after a traumatic hip fracture between the highest- and lowest-spending hospital quintiles. The majority of this variation is explained by choice of PAC discharge setting, with the highest-spending hospitals utilizing inpatient rehabilitation 4 times more often than the lowest-spending hospitals. Further work is needed to understand the influence of PAC setting and services on patient-reported outcomes before differences in value can be explored.

## ACKNOWLEDGMENTS

J.R.M., A.H.C.-N., and M.R.H. had full access to all the data in the study and take responsibility for the integrity of the data and the accuracy of the data analysis.

## Supplementary Material


