# Supplementary material for: Sources of Postacute Care Episode Payment Variation After Traumatic Hip Fracture Repair Among Medicare Beneficiaries: Cross-Sectional Retrospective Study
Source: Ann Surg Open. 2022 Nov 7;3(4):e218. doi: 10.1097/AS9.0000000000000218 (PMC10406045; doi:10.1097/AS9.0000000000000218)
Supplement: Supplementary file 1 [file as9-3-e218-s001.pdf]

Supplemental Data File 1. Table that includes the International Classification of Diseases 9 and 10 codes and descriptors used to create the traumatic hip fracture injury cohort.

ICD-9 or ICD-10 Code
82000, 82001, 82002, 82003, 82009, 8201, 82010, 82011, 82012, 82013, 82019, 82020, 82021, 82022, 82030, 82031, 82032, 8208, 82080, 8209, 82090, S72001A, S72001B, S72001C, S72002A, S72002B, S72002C, S72009A, S72009B, S72009C, S72011A, S72011B, S72011C, S72012A, S72012B, S72012C, S72019A, S72002B, S72002C, S72009A, S72009B, S72009C, S72011A, S72011B, S72011C, S72012A, S72012B, S72012C, S72019A, S72019B, S72019C, S72031A, S72031B, S72031C, S72032A, S72032B, S72032C, S72033A, S72033B, S72033C, S72034A, S72034B, S72034C, S72035A, S72035B, S72035C, S72036A, S72036B, S72036C, S72041A, S72041B, S72041C, S72042A, S72042B, S72042C, S72043A, S72043B, S72043C, S72044A, S72044B, S72044C, S72045A, S72045B, S72045C, S72046A, S72046B, S72046C, S72051A, S72051B, S72051C, S72052A, S72052B, S72052C, S72059A, S72059B, S72059C, S72061A, S72061B, S72061C, S72062A, S72062B, S72062C, S72063A, S72063B, S72063C, S72064A, S72064B, S72064C, S72065A, S72065B, S7206SC, S72066A, S72066B, S72066C, S72091A, S72091B, S72091C, S72092A, S72092B, S72092C, S72099A, S72099B, S72099C, S72101A, S72101B, S72101C, S72102A, S72102B, S72102C, S72109A, S72109B, S72109C, S72111A, S72111B, S72111C, S72112A, S72112B, S72112C, S72113A, S72113B, S72113C, S72114A, S72114B, S72114C, S72115A, S72115B, S72115C, S72116A, S72116B, S72116C, S72121A, S72121B, S72121C, S72122A, S72122B, S72122C, S72123A, S72123B, S72123C, S72124A, S72124B, S72124C, S72125A, S72125B, S72125C, S72126A, S72126B, S72126C, S72131A, S72131B, S72131C, S72132A, S72132B, S72132C, S72133A, S72133B, S72133C, S72134A, S72134B, S72134C, S72135A, S72135B, S72135C, S72136A, S72136B, S72136C, S72141A, S72141B, S72141C, S72142A, S72142B, S72142C, S72143A, S72143B, S72143C, S72144A, S72144B, S72144C, S72145A, S72145B, S72145C, S72146A, S72146B, S72146C, S7221XA, S7221XB, S7221XC, S7222XA, S7222XB, S7222XC, S7223XA, S7223XB, S7223XC, S7224XA, S7224XB, S7224XC, S7225XA, S7225XB, S7225XC, S7226XA, S7226XB, S7226XC

Abbreviation: ICD, International Classification of Diseases.
