# Supplementary material for: Sources of Postacute Care Episode Payment Variation After Traumatic Hip Fracture Repair Among Medicare Beneficiaries: Cross-Sectional Retrospective Study
Source: Ann Surg Open. 2022 Nov 7;3(4):e218. doi: 10.1097/AS9.0000000000000218 (PMC10406045; doi:10.1097/AS9.0000000000000218)
Supplement: Supplementary file 2 [file as9-3-e218-s002.pdf]

Supplemental Data File 2. Patient, Injury, and Hospital Characteristics by 90-day Mean PAC episode spending quintile. Hospitals were placed into quintiles based on their inflation-adjusted mean PAC spending per episode. Data presented as percentage (%) unless otherwise noted.

	**Q1 (Lowest)**	**Q5 (Highest)**	***P* value**
General Characteristics			
Hospitals, n	416	415	
Patients, n	22,000	23,171	
Inpatient episode spending, mean (SD)	$15,148 ($2,506)	$19,049 ($4,332)	<0.001
Hospital length of stay, mean (SD)	4.9 (0.9)	5.3 (1.0)	<0.001
PAC episode spending, mean (SD)	$17,681 ($1,749)	$31,831 ($4,276)	<0.001
Readmission within 90 days	19.0	25.3	<0.001
Patient & Injury Characteristics			
Male sex	27.1	17.8	0.12
Age, mean (SD)	83.2 (1.8)	83.3 (1.8)	0.04
Race			
White	95.2	86.7	<0.001
Black	2.5	4.5	
Other	2.3	8.8	
Injury severity score			
9	91.5	91.7	0.50
10	8.3	8.0	
11	0.2	0.3	
Elixhauser comorbidity index, mean (SD)	7.2 (1.7)	7.3 (1.6)	0.79
Experienced inpatient complication	18.9	20.9	0.01
Hospital Characteristics			
Region			
Midwest	30.0	11.6	<0.001
Northeast	10.6	26.7	
South	45.4	28.4	
West	13.9	33.3	
Beds			

<200	60.3	37.1	<0.001
200-349	23.1	31.8	
350-499	7.2	16.6	
≥500	9.4	14.5	
Teaching	60.8	72.8	0.003
Urban	93.3	98.6	<0.001
Profit status			
For profit	14.7	19.5	0.13
Non-profit	74.5	67.0	
Other	10.8	13.5	
Critical access	2.6	1.4	0.62

Abbreviation: PAC, post-acute care; SD, standard deviation.
