# Supplementary material for: Sources of Postacute Care Episode Payment Variation After Traumatic Hip Fracture Repair Among Medicare Beneficiaries: Cross-Sectional Retrospective Study
Source: Ann Surg Open. 2022 Nov 7;3(4):e218. doi: 10.1097/AS9.0000000000000218 (PMC10406045; doi:10.1097/AS9.0000000000000218)

Supplemental Data File 3. Figure that illustrates hospital-level mean risk-adjusted inpatient payments and mean risk-adjusted total 90-day PAC payments.

There was no significant correlation between payments (ρ = -0.012, 95% CI -0.057 to 0.033). PAC and index hospitalization spending data is inflation adjusted and price standardized. PAC data is adjusted for case mix, hospital factors, and readmissions. Index hospitalization is adjusted for case mix and hospital factors.

Abbreviation: PAC, post-acute care

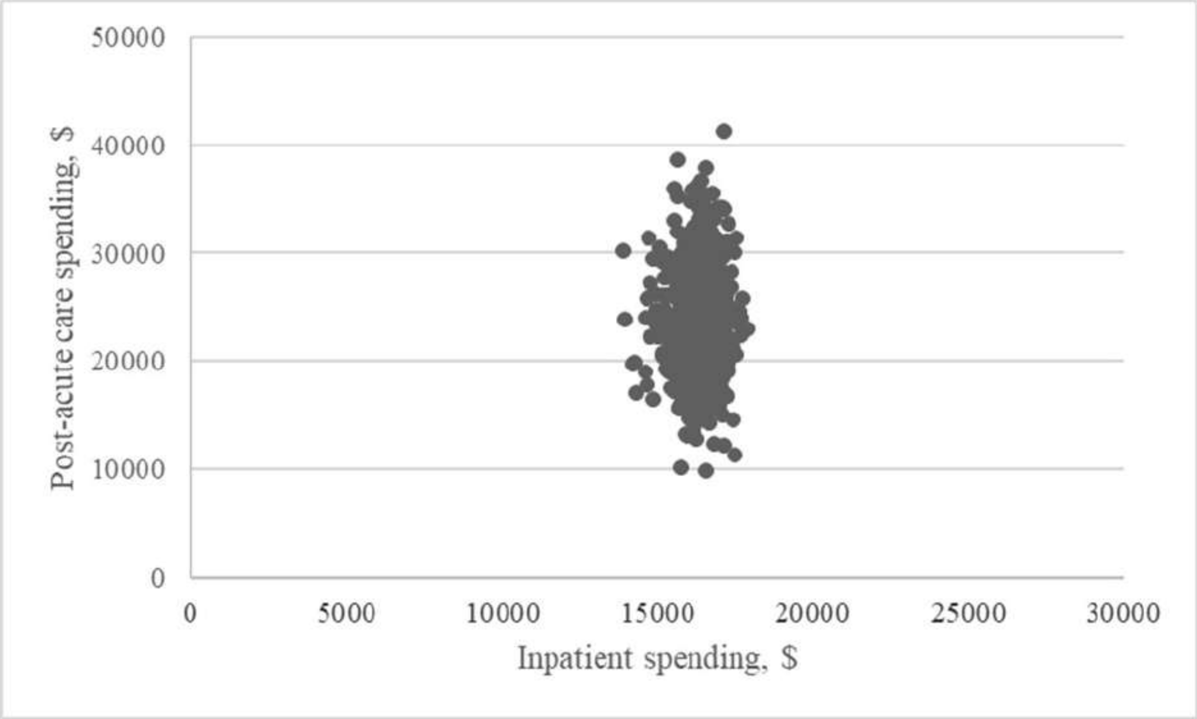

Supplement: Supplementary file 3 [file as9-3-e218-s003.pdf]
